# Thrombectomy for medium-sized cerebral vessel occlusion: Size does matter

**DOI:** 10.1093/esj/23969873251376862

**Published:** 2026-01-01

**Authors:** Pekka Virtanen, Silja Räty, Liisa Tomppo, Nina Brandstack, Erno Peltola, Tatu Kokkonen, Mikko Sillanpää, Daniel Strbian

**Affiliations:** Department of Radiology, Helsinki University Hospital and University of Helsinki, Helsinki, Finland; Department of Neurology, Helsinki University Hospital and University of Helsinki, Helsinki, Finland; Department of Neurology, Helsinki University Hospital and University of Helsinki, Helsinki, Finland; Department of Radiology, Helsinki University Hospital and University of Helsinki, Helsinki, Finland; Department of Radiology, Helsinki University Hospital and University of Helsinki, Helsinki, Finland; Department of Radiology, Helsinki University Hospital and University of Helsinki, Helsinki, Finland; Department of Radiology, Helsinki University Hospital and University of Helsinki, Helsinki, Finland; Department of Neurology, Helsinki University Hospital and University of Helsinki, Helsinki, Finland

**Keywords:** Ischaemic stroke, thrombectomy, vessel size, outcome%

## Abstract

**Introduction:**

Randomised controlled trials comparing endovascular thrombectomy (EVT) to medical treatment in patients with medium vessel occlusion (MeVO) suggested neutrality or futility of EVT. We studied whether the size difference between thrombectomy device and the occluded vessel influenced MeVO outcomes.

**Patients and methods:**

This was a retrospective single-centre observational study comprising EVT-treated patients with occlusion of the M2 branch of the middle cerebral artery on digital subtraction angiography. The diameter of the occluded M2 was measured and compared to the manufacturer’s recommendation for the minimal vessel size. Based on this device-to-vessel size ratio, we divided the patients into three groups: A) ratio ⩽1.0 (device smaller or equals the vessel size), B) 1.0 < ratio ⩽ 1.2 (device larger, difference ⩽20%), and C) ratio >1.2 (device larger, significant difference >20%). The primary outcomes were futility (3-month modified Rankin scale 5 or 6) and symptomatic intracranial haemorrhage (sICH).

**Results:**

In the cohort of 146 patients (median age 73; 47.3% women), 58.9% were in group A, 13.7% in group B and 27.4% in group C. Patients in group C had more frequently sICH (20.0%) compared to group A (7.0%) and group B (5.0%), and the highest futility rate (34.2% vs 17.3% vs 25.0%, respectively). In the adjusted analyses, belonging to the group C was associated with sICH (OR 3.32 [1.04–10.64]) and mRS 5-6 (OR 2.84 [1.09–7.37]).

**Discussion and conclusions:**

The size of the thrombectomy device relative to the size of the occluded vessel is associated with haemorrhagic complications and futile outcomes.

## Graphical Abstract

**Graphical abstract:**
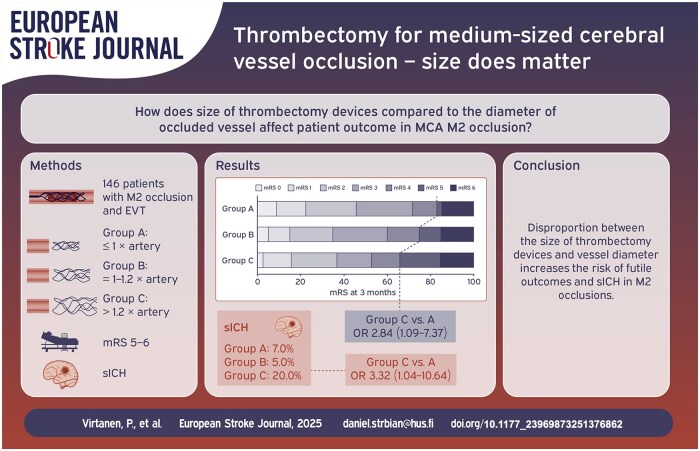

## Introduction

Endovascular thrombectomy (EVT) is an evidence-based therapy for large vessel occlusions (LVOs), but most of the trials included only a few patients with medium vessel occlusions (MeVOs). An individual patient-level meta-analysis of these trials^1^ suggested benefit for those with occlusion of the dominant M2 branch of the middle cerebral artery (MCA), however, non-randomised studies reported best medical treatment (BMT) to be at least as good as the EVT.^2^ The same was found for the posterior cerebral artery.^3^

As a response to the clinical equipoise, the results of three randomised controlled trials (RTC) on EVT versus BMT for patients with MeVOs were reported in 2025. Two did not find difference between the treatments,^4,5^ whereas the last one^6^ reported (at the International Stroke Conference 2025) worse outcomes in the EVT arm. There is a considerable difference in vessel size between LVOs and MeVOs. The size of the occluded vessel is typically not known nor easily measurable, and the device (aspiration catheter or stent retriever) selection is done at the discretion of the interventionalists.

In the current study, we analysed vessel metrics in patients with occlusions in the M2 segment of the MCA; aiming to guide the optimal device selection for procedural safety when operating in smaller vessels. We hypothesised that the size of the thrombectomy instrument in relation to the diameter of the occluded M2 branch is associated with functional outcomes and frequency of haemorrhagic complications.

## Patients and methods

This was a single-centre retrospective observational study from the Helsinki University Hospital on patients with acute ischaemic stroke between January 2018 and December 2021. We selected all patients with an occlusion of the M2 branch of the MCA on initial digital subtraction angiography (DSA). The decision to perform further EVT for M2 occlusion was made on an individual basis by the treating neurologist. IVT was administered when indicated. EVT was performed by a specialised interventional radiologist or a neurointerventional radiologist using third-generation stent retrievers along with other modern endovascular techniques (direct stent retrieval, direct aspiration, or combined method). Clinical data were obtained from electronic medical records. The study is reported according to the recommended guidelines.^7^

Anatomy of M2 segment has some variability – both in the number of divisions and in division dominance.^8^ In this work, we used existing classification based on CT angiography (CTA) in addition to DSA.^9^ Occluded M2 vessels were categorised into superior and inferior branches and dominant, co-dominant or non-dominant based on perfusion images when available (85 patients) or using visual assessment from angiographic imaging.^4^

**Figure 1.**
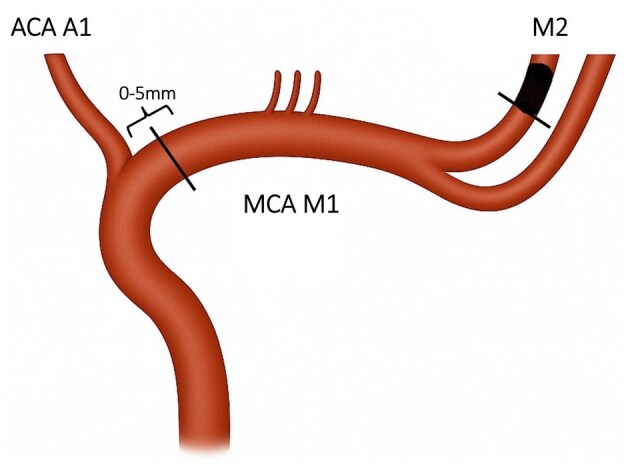
Visualisation for measurements of diameters of the occluded M2 and ipsilateral M1 branches of the middle cerebral artery occlusion The figure shows the location for measurements of vessel diameter: M1 was measured within 5 mm from the A1 segment of the anterior cerebral artery. ACA: anterior cerebral artery; MCA: middle cerebral artery.

The bilateral proximal M1 segments and the occluded M2 segment diameters were measured from DSA. We performed measurements also on baseline CTA, which were made perpendicular to the artery lumen (a) on cross sectional plane and (b) from maximum intensity projection (MIP) images. The proximal diameter of the M1 segment was measured within 0–5 mm from the A1 branch of the anterior cerebral artery, and the occluded M2 was measured as proximally as possible to the occlusion (Figure 1). The measurements were made from ipsi- and contralateral M1 segments to account for possible atherosclerotic changes, which were defined as visible calcified atherosclerotic plaque, typical atherosclerotic morphology and lesion stability in follow up imaging. Intracranial stenosis was graded as absent, minimal or over 50% (visual assessment). Voxel size in CTA reformats was 0.6 mm in over 95% of cases, and the rest varied up to 1.5 mm. A second experienced neuroradiologist (NB) repeated the measurements of M1 and M2 diameters in CTA of 50 patients to obtain interobserver variability.

The Acute Stroke Prognosis Early CT Score (ASPECTS)^10^ was calculated from the baseline and 24-h non-contrast CT. Virtual native images from dual energy CT were used to evaluate contrast enhancement. The reperfusion was classified with modified Medium-Vessel-Occlusion-Expanded Thrombolysis in Cerebral Infarction (MeVo-M-TICI).^11^

All devices were recorded. The manufacturer-recommended minimum vessel diameter was obtained from the manufacturer’s own stated values, as indicated in the product datasheet and package insert and outer diameter for aspiration catheters. This was compared to the diameter of the occluded M2 vessel from DSA. We calculated the absolute difference between the device recommendation and the vessel diameter, and also the device-to-vessel size ratio. Based on the ratio, patients were divided into three groups: (A) ratio ⩽ 1.0, meaning that device was smaller or equalled size of the occluded M2, (B) 1.0 < ratio ⩽ 1.2: size of the device was larger than occluded M2 with mild-to-moderate difference up to 20%, and (C) ratio > 1.2: size of the device was significantly larger than occluded M2 with difference more than 20%.

All radiological data were analysed on a picture archiving and communication system workstation (PACS, AGFA and Siemens) and by using multiplanar reconstruction software or third party software (Vitrea, Vital Imaging).

The co-primary outcomes were futility, defined as severe dependency or death (a modified Rankin scale (mRS) score of 5 or 6) at 3 months and symptomatic intracranial haemorrhage (sICH) according to the European Cooperative Acute Stroke Study II (ECASS II) classification.^12^ Three-month mRS was determined by a certified stroke neurologist based on information gathered from a phone interview and electronic patient records. Secondary outcomes comprised MeVo-M-TICI after endovascular procedure, National Institutes of Health Stroke Scale (NIHSS) at 24 h, any haemorrhage and mortality at 3 months.

### Statistical analyses

For continuous variables, median and interquartile range values were calculated, and the Kruskal-Wallis ANOVA compared baseline differences. For categorical variables, percentage proportions were calculated and compared with the χ^2^ method. Regarding the primary outcomes (mRS 5-6 and sICH), models of binary logistic regression were constructed, adjusted for known strong predictors: age and pre-EVT NIHSS. We could not force more variables into the model, as the ‘1 in 10’ rule for logistic regression suggests having at least 10 (outcome) events for every predictor variable (32 patients reached the primary outcome). For sensitivity analysis, we merged groups A and B. The Pearson correlation coefficient was calculated for DSA versus CTA measurements. Interclass correlation coefficient (ICC, average measures) was obtained to quantitate the extent of agreement between raters. *p* Values of <0.05 (two-tailed) were considered significant.

## Results

Among 954 patients who underwent DSA during the study period, 182 had an occlusion located in the M2 branch of the MCA. For 34 of them, the treating neurologist decided not to proceed to EVT. Two additional patients were excluded from the analysis, as we could not measure their vessel diameters. Hence, our final cohort included 146 patients, 69 (47.3%) of whom were women. They had a median age of 73 (65–80) years, baseline NIHSS of 10 (7–16), last-seen-well (LSW)-to-arrival time of 160 (76–354) min and puncture to arrival time of 59 (49–78) min.

Based on the device-to-vessel size ratio, 86 (58.9%) were in group A (device smaller or equals the vessel size), 20 (13.7%) in group B (device larger, difference ⩽20%) and 40 (27.4%) in group C (device larger, significant difference >20%). Their baseline characteristics are outlined in Table 1. Briefly, the patients in group C were more frequently women and had non-significantly longer LSW delays-to-arrival. The patients in group B had a higher proportion of history of atrial fibrillation and cardioembolic aetiology of the index stroke. We had only five cases of intracranial atherosclerotic disease (all had stenosis <50%).

**Table 1.** Baseline characteristics.

Variable	Groups based on the device-to-vessel size ratio			*p*-Value
	A (⩽1, *n* = 86)	B (⩽1.2, *n* = 20)	C (>1.2; *n* = 40)	
Age, years	75 (65–80)	75 (68–79)	70 (60–78)	0.20
Female sex, *n* (%)	34 (39.5)	10 (50.0)	25 (62.5)	0.051
pre-stroke mRS, points	0 (0–0)	0 (0–0)	0 (0–0)	1.00
LSW to arrival, min	150 (67–351)	122 (63–319)	236 (105–357)	0.46
Arrival to puncture, min	60 (49–74)	60 (51–83)	65 (50–80)	0.69
NIHSS prior to EVT, points	10 (6–15)	13 (7–16)	11 (6–17)	0.47
ASPECTS, points	9 (8–10)	10 (8–10)	10 (8–10)	0.61
IVT, *n* (%)	42 (48.8)	8 (40.0)	17 (42.5)	0.73
*History of*				
Ischaemic stroke, *n* (%)	13 (15.1)	2 (10.0)	9 (22.5)	0.41
TIA, *n* (%)	3 (3.5)	1 (5.0)	2 (5.0)	1.00
CAD, *n* (%)	13 (15.1)	2 (10.0)	4 (10.0)	0.73
Atrial fibrillation, *n* (%)	22 (25.6)	11 (55.0)	12 (30.0)	**0.04**
Hypertension, *n* (%)	43 (50.0)	10 (52.6)	24 (60.0)	0.61
Diabetes, *n* (%)	12 (14.0)	3 (15.0)	7 (17.5)	0.95
*TOAST*				
LAA, *n* (%)	17 (19.8)	1 (5.0)	9 (22.5)	0.18
CE, *n* (%)	50 (58.1)	17 (85.0)	22 (55.0)	
Other, *n* (%)	6 (7.0)	0	1 (2.5)	
Insufficient/unknown, *n* (%)	13 (15.1)	2 (20.0)	8 (20.0)	

mRS: modified Rankin Scale; LSW: last seen well; NIHSS: National Institutes of Health Stroke Scale; EVT: endovascular thrombectomy; ASPECTS: Acute Stroke Prognosis Early CT Score; IVT: intravenous thrombolysis; TIA: transient ischaemic attack; CAD: coronary artery disease; LAA: large artery disease; CE: cardioembolism.

Unless otherwise stated, data are presented as median and IQR. Significant *p-*values are bolded.

While the diameters of ipsilateral and contralateral M1 branches were very similar among the three groups, the size of the M2 branch was smallest in group C (Table 2). This group also had non-significantly higher proportions of non-dominant M2 occlusions, and the largest size of used devices. In line, the difference between the size of the device and vessel among the groups was statistically significant. Inferior branch occlusion was represented similarly between groups (Group A 38.4%, Group B 25% and Group C 27.5%, *p* = 0.36) There was no difference regarding the final MeVo-M-TICI 2b-3 or 2c-3 (Table 3). The majority of the patients in all groups (up to 70%) had 1–2 passes; however, at least 4 passes were performed more frequently in group C (Table 2). Aspiration was very seldom used as the only technique.

**Table 2.** CTA and DSA characteristics.

Variable	Groups based on the device-to-vessel size ratio			*p*-Value
	A (⩽1, *n* = 86)	B (⩽1.2, *n* = 20)	C (>1.2; *n* = 40)	
*CTA*				
Ipsilateral M1, mm	2.40 (2.10–2.60)	2.45 (2.10–2.55)	2.40 (2.10–2.50)	0.78
Contralateral M1, mm	2.40 (2.10–2.60)	2.40 (2.15–2.60)	2.30 (2.10–2.50)	0.42
Occluded M2, mm	1.70 (1.60–2.00)	1.60 (1.50–2.00)	1.60 (1.40–1.80)	**0.02**
*DSA*				
MeVo-M-TICI 0–1, *n* (%)	86 (100)	20 (100)	40 (100)	1.00
Ipsilateral M1, mm	2.40 (2.20–2.50)	2.40 (2.15–2.60)	2.30 (2.20–2.50)	0.85
Occluded M2, mm	1.70 (1.60–1.90)	1.65 (1.35–2.15)	1.60 (1.40–1.75)	<**0.01**
Non-dominant M2, *n* (%)	33 (38.8)	7 (35.0)	21 (52.5)	0.30
Size of device, mm	1.50 (1.50–1.50)	1.81 (1.50–2.50)	2.50 (2.50–2.50)	<**0.001**
Difference, mm	−0.20 (−0.40 to −0.10)	0.20 (0.10–0.20)	0.80 (0.70–1.00)	<**0.001**
*EVT technique*				
Stent-retriever, *n* (%)	78 (90.7)	16 (80.0)	32 (80.0)	0.10
Aspiration, *n* (%)	3 (3.5)	1 (5.0)	0	
Combined, *n* (%)	5 (5.8)	3 (15.0)	8 (20.0)	
*Number of attempts, n (%)*				
1	43 (50.0)	8 (40.0)	12 (30.0)	0.16
2	20 (23.3)	5 (25.0)	13 (32.5)	
3	16 (18.6)	4 (20.0)	5 (12.5)	
4 and more	7 (8.1)	3 (15)	10 (25)	

CTA: computed tomography angiography; DSA: digital subtraction angiography; MeVo-M-TICI: Medium-Vessel-Occlusion-Expanded Thrombolysis in Cerebral Infarction; EVT: endovascular thrombectomy.

Unless otherwise stated, data are presented as median and IQR. Significant *p*-values are bolded.

**Table 3.** Unadjusted outcomes.

Variable	Groups based on the device-to-vessel size ratio			*p*-Value
	A (⩽1, *n* = 86)	B (⩽1.2, *n* = 20)	C (>1.2; *n* = 40)	
*MeVo-M-TICI post EVT*				
0-2A, *n* (%)	31 (36.0)	6 (30.0)	15 (37.5)	0.88
2B-2C-3, *n* (%)	55 (64.0)	14 (70.0)	25 (62.5)	
2C-3, *n* (%)	39 (45.3)	10 (50.0)	15 (37.5)	0.59
*24 h*				
NIHSS, points	7 (3–14)	10 (4–14)	10 (5–19)	0.24
ASPECTS, points	7 (5–8)	7 (7–9)	6 (5–7)	0.06
*Haemorrhage*				
any ICH, *n* (%)	36 (41.9)	7 (35.0)	19 (47.5)	0.60
ECASS II, *n* (%)	6 (7.0)	1 (5.0)	8 (20.0)	**0.04**
SITS, *n* (%)	3 (3.5)	0	5 (12.5)	0.06
*3 months*				
mRS 5-6, *n* (%)	14 (17.3)	5 (25.0)	13 (34.2)	0.12
mortality, *n* (%)	12 (14.8)	3 (15.0)	6 (15.8)	1.00

MeVo-M-TICI: Medium-Vessel-Occlusion-Expanded Thrombolysis in Cerebral Infarction; EVT: endovascular thrombectomy; NIHSS: National Institutes of Health Stroke Scale; ASPECTS: Acute Stroke Prognosis Early CT Score; ICH: intracerebral haemorrhage; ECASS II: European Cooperative Acute Stroke Study II; SITS: safe Implementation of Treatments in Stroke; mRS: modified Rankin Scale.

Unless otherwise stated, data are presented as median and IQR. Significant *p*-values are bolded.

In general, females had smaller vessels than males (Supplemental Table 1). We found an excellent correlation between DSA and CTA measurements (0.923, *p* < 0.001). For CTA, measurements from perpendicular reformat images were practically identical to MIP images (data not shown). Interrater agreement was excellent for M1 (0.884, *p* < 0.001) and M2 (0.879, *p* < 0.001) diameter measurements.

**Figure 2.**
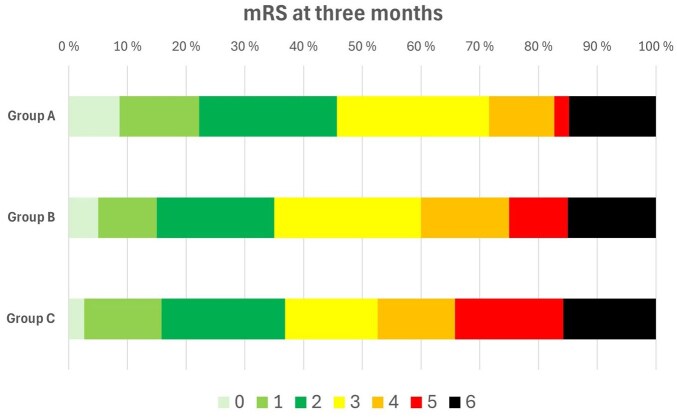
Distribution of 3-month modified Rankin Scale (mRS).

Patients in group C had more frequently sICH (20.0%) compared to group A (7.0%) and group B (5.0%) (Table 3). They also had the biggest proportion of mRS 5-6 at 3 months (34.2%) compared to 17.3% and 25.0%, respectively (Figure 2). In the adjusted analyses, belonging to group C was independently associated with higher odds of sICH (OR 3.32 [95% CI 1.04–10.64], *p* = 0.03) and futile outcome (OR 2.84 [95% CI 1.09–7.37], *p* = 0.04) compared to group A (Table 4). For the secondary outcomes, there were no significant differences between the groups in the unadjusted (Table 3) and adjusted analysis (data not shown).

**Table 4.** Adjusted outcome analyses.

Variable	OR	95% CI	*p*-Value
*sICH (ECASS II)*			
Age, per 1-year change	1.01	0.96–1.06	0.78
NIHSS, per 1-point change	1.02	0.94–1.12	0.59
Device-to-vessel ratio	Reference: ratio ⩽ 1.0		
1.0 > Device-to-vessel ratio ⩽ 1.2	0.67	0.08–5.94	0.72
Device-to-vessel ratio > 1.2	3.32	1.04–10.64	**0.04**
*mRS 5-6*			
Age, per 1-year change	1.04	1.00–1.09	**0.049**
NIHSS, per 1-point change	1.10	1.02–1.18	**0.01**
Device-to-vessel ratio	Reference: ratio ⩽ 1.0		
1.0 > Device-to-vessel ratio ⩽ 1.2	1.41	0.42–4.72	0.58
Device-to-vessel ratio > 1.2	2.84	1.09–7.37	**0.03**

sICH: symptomatic intracerebral haemorrhage; ECASS II: European Cooperative Acute Stroke Study II; NIHSS: National Institutes of Health Stroke Scale; mRS: modified Rankin Scale; OR: odds ratio; CI: confidence interval.

Very similar results in case the ratio was entered as a non-categorical parameter. Significant p-values are bolded.

In the sensitivity analysis, we merged groups A and B and observed very similar adjusted results disfavouring group C: (a) OR 3.56 (1.16–10.88), *p* = 0.03, for sICH, and (b) OR 2.61 (1.06–6.45), *p* = 0.04, for futile outcome.

### Additional outcome analyses

Because the vessel size was smaller in females compared to males and due to an uneven distribution of sexes among the groups, we tested adjusted effect of sex on outcome. None was observed (data not shown).

In the Supplemental Material, we provide data on 34 patients, in whom the treating physician made a decision not to proceed to further EVT (Supplemental Table 2). These patients had very similar vessel metrics to group C (Supplemental Table 3), but their outcome was comparable to group A (Supplemental Table 4 and Supplemental Figure 1).

## Discussion

We found that using thrombectomy devices outside manufacturer-recommended vessel diameter in M2 occlusions was associated with increased rates of sICH and futile functional outcomes. Second, the patients treated with appropriately sized instruments had outcomes comparable to M2-occlusion patients who did not undergo EVT. These findings are important considering recently published RCTs^4,5^and non-randomised studies,^13^ contributing to the debate why EVT was not superior to BMT.

ESCAPE-MeVO reported increased mortality of EVT compared to BMT (13.3% vs 8.4%) and a higher incidence of serious adverse events (33.9% vs 25.7%, respectively). Another MeVO RCT^6^ was terminated prematurely due to futility in the EVT arm. Compared to the DISTAL trial, the independent functional outcome was somewhat lower in our study (~55% vs 46%), however, we included strictly patients with M2 occlusions unlike DISTAL.^4^ In DISTAL, 71.7% reached a mTICI score ⩾ 2b, and 65.1% in our study. Our rates of mTICI ⩾ 2c and functional independence were akin to findings of a recent multicentre observational study.^13^ Interestingly, some early studies failed to demonstrate an association between recanalisation and outcomes in M2 occlusions, implying a higher risk of futile recanalisation in MeVOs.^14^

Most modern stent retrievers are laser-cut or braided. Laser-cut designs, used exclusively in our study, may exert higher radial force in smaller vessels, raising concerns about endothelial injury.^15,16^ The recommended minimum vessel diameter is based on factors such as radial force, and device use within these limits is considered safe by manufacturer. However, data on safety in smaller vessels, like those in our study, may be limited. While sufficient force is needed for thrombus engagement, excessive force or oversized devices can cause complications. In our cohort, the median M2 occlusion diameter was 1.6–1.7 mm, and few stent retrievers were approved for use in vessels of this size at the time. Moreover, when deploying a stent retriever, its distal end may temporarily end up in a branch of a smaller diameter.

The selection of stent retriever, aspiration or combined approach is not guided by evidence-based data. One meta-analysis reported more frequent excellent outcomes (despite comparable recanalisation rates) after aspiration for distal M2 occlusions,^17^ while others found aspiration to be less efficient.^18^ Higher subarachnoid haemorrhage rates have been observed with stent retrievers and combined techniques compared to aspiration alone, although this did not affect clinical outcomes.^19^ Most of our patients underwent EVT with stent retrievers, which was also a requirement for the first thrombus-retrieval attempt (with or without aspiration) in the MeVO ESCAPE trial.^5^ In the DISTAL trial, aspiration alone was performed in 15.7%.^4^

With multiple anatomical variations, sharply angulated and tortuous M2 segments may pose technical challenges and increase the risk of complications.^20^ While dominant M2 locations were excluded from the DISTAL trial, MeVO ESCAPE included both proximal and distal M2 occlusions. In our study, 42% of occlusions were in the non-dominant M2, which was non-significantly over-represented in group C. In general, distal M2 segments are less robust and more loosely attached to parenchyma, increasing risks of displacement or perforator injury during EVT.^21^

This becomes more relevant with increasing EVT passes, which are linked to higher rates of futile recanalisation.^22^ Indeed, the highest proportion of 4+ passes was in group C. The impact of anaesthesia type (and movement of patients) on distal occlusions remains unclear, but most of our EVTs were done under conscious sedation to avoid delays.

We observed that females had significantly smaller vessels than males, and that sex was not evenly distributed among the groups. Nonetheless, sex did not have any effect on the outcome.

Measurements from the DSA are less prone to artefacts, as CTA measurements can have small errors due to a partial volume effect. In addition to DSA, we also measured diameters from the CTA. Spatial resolution of CTA is a critical determinant of measurement accuracy. Indeed, over or underestimation of the diameter can be pronounced in small vessels, where diameter approaches the voxel size.^23^ This can be mitigated with high resolution CT scanners and advanced reconstruction algorithms. Thus, it is reassuring that voxel size in our CTA reformats was 0.6 mm in over 95% of cases.

We found an excellent correlation between DSA and CTA measurements (including those from MIP). Yet, manual measuring can be time consuming. Measurements from MIP images or utilising semi- or fully automated solutions or machine learning-based methods^24^ could provide rapid measurements without delaying EVT. As we did not have a reliable and accurate automated solution, this could be tested in future.

Further research could explore branching angles of M1/M2, as large ICA/M1 angles were suggested to affect M2 thrombectomy success.^25^ Modern-design segmented stent retrievers reduce clot fragmentation compared to older collapsed types, especially in tortuous vessels.^26^ Pre-procedural device matching to vessel size using 3D rotational angiography has shown promise – one study with a median vessel diameter of 1.71 mm (similar to ours) found no sICH, though asymptomatic subarachnoid haemorrhage increased as vessel size decreased.^27^ Automated solutions could also be used to analyse vessel tortuosity in the future. Finally, motion correction algorithms and dual-energy or photon counting CTA could improve measurement accuracy.

Due to the retrospective observational design and limited sample size, the study possesses the risk of confounding bias. The results were obtained from a single centre, and more studies are needed to improve the generalisation. Our study included only M2, however, it was the most common occlusion location in the RCTs and observational studies on MeVO, and the possibility to measure vessel size is more feasible for this location compared to more distal ones. The outcomes were analysed based on the generic type of device, not by the manufacturer. Nonetheless, the full list is available in the Supplemental Methods. Our observations reflect the devices available at the time.

## Conclusion

Careful consideration is important when planning EVT for patients with occlusion in the M2 branch of the MCA. The size of the device related to the diameter of the occluded M2 branch was associated with both haemorrhagic complications and futile functional outcome. Whether this was the case in the MeVO RCTs remains to be addressed.

## Supplementary Material

supplementary_files_23969873251376862

## Data Availability

Anonymised data are available upon reasonable request to the corresponding author following national legislation.
